# Graphene Hybrid Materials for Controlling Cellular Microenvironments

**DOI:** 10.3390/ma13184008

**Published:** 2020-09-10

**Authors:** Cheol-Hwi Kim, Tae-Hyung Kim

**Affiliations:** 1 School of Integrative Engineering, Chung-Ang University, Seoul 06974, Korea; sorksk@cau.ac.kr; 2 Integrative Research Centre for Two-Dimensional Functional Materials, Institute of Interdisciplinary Convergence Research, Chung-Ang University, Seoul 06974, Korea

**Keywords:** cellular microenvironment, graphene, three-dimensional cell culture, tumor modeling, stem cell differentiation

## Abstract

Cellular microenvironments are known as key factors controlling various cell functions, including adhesion, growth, migration, differentiation, and apoptosis. Many materials, including proteins, polymers, and metal hybrid composites, are reportedly effective in regulating cellular microenvironments, mostly via reshaping and manipulating cell morphologies, which ultimately affect cytoskeletal dynamics and related genetic behaviors. Recently, graphene and its derivatives have emerged as promising materials in biomedical research owing to their biocompatible properties as well as unique physicochemical characteristics. In this review, we will highlight and discuss recent studies reporting the regulation of the cellular microenvironment, with particular focus on the use of graphene derivatives or graphene hybrid materials to effectively control stem cell differentiation and cancer cell functions and behaviors. We hope that this review will accelerate research on the use of graphene derivatives to regulate various cellular microenvironments, which will ultimately be useful for both cancer therapy and stem cell-based regenerative medicine.

## 1. Introduction

Cells experience growth within the cell microenvironment [1,2]. Specifically, the cellular microenvironment contains the extracellular matrix and is composed of various types of proteins. [3,4]. These have a profound effect on the future of the cell through interaction with the cell. These interactions are integral for engineering tissues or organs and are closely related to regenerative medicine. Control of the cellular microenvironment implies control of cell proliferation and differentiation. By “controlling cellular microenvironments”, many researchers study technologies that can fundamentally be used to treat certain diseases, including stem cell treatments and various drugs or materials that promote cellular regeneration of damaged human tissues [5,6,7,8,9,10,11,12]. In addition, controlling the cellular microenvironment has been spotlighted in the field of cancer research, particularly regarding attempts to mimic tumors that develop in the human body [13,14,15]. The research value of three-dimensional (3D) cell culture methods is preferred because 3D cell cultures mimic tumors more closely compared to conventional two-dimensional (2D) cell cultures [16,17,18,19]. Among related studies on the control of cellular microenvironments, research into cell culture methods to arbitrarily limit the growth range of cells to create a 3D cell cluster of the desired type has been actively pursued, and through this, attempts to replace clinical trials are continuously being conducted.

ECMs, which are part of the cellular microenvironment, are primarily composed of proteoglycan and fibrous proteins. In particular, fibrous protein contributes to cell adhesion mechanisms and tissue development and plays a pivotal role in the aforementioned control of the cellular microenvironment [20,21,22,23]. While fibrous proteins (collagen, fibronectin, chitosan, among others) include all the properties common to proteins, they also include the property of degeneration in extreme environments such as high temperatures [24,25,26,27,28]. Because of those properties, many researchers have been limited in some applications when studying fibrous proteins.

Graphene is one of the most popular nanomaterials used in various studies mostly due to its unique physicochemical properties [29]. Generally, graphene is known to possess high physical and chemical resistance [30,31,32], very high electrical conductivity, and excellent strength and thermal conductivity [33,34,35,36]. Graphene oxide (GO), a well-known graphene derivative, is particularly attractive for biomedical studies because of the presence of oxygen groups on both in-plane and edge structure. These hydrophilic groups of GO include hydroxyl, carbonyl, carboxyl, and carboxylate moieties, and they enable increased interaction with proteins (e.g., growth factors, ECM proteins) through electrostatic, covalent, and hydrogen bonding. Such enhanced interactions between GO and proteins are known to strongly improve cell adhesion on an artificial surface [37,38,39]. In addition, GO is also known to be highly adhesive to the phospholipid bilayer of the cell membrane and induces more effective cell adhesion. Specifically, the phosphate and polar head groups existing in the outermost layer of the cell membrane show a high affinity toward the hydrophilic functional groups of GO via electrostatic and hydrophilic interactions [40,41]. Moreover, since graphene derivatives, including GO, are carbon-based materials, they are known to be less toxic to cells and tissues, and thus are attractive biocompatible materials for the use in regenerative medicine [42,43,44].

**Figure 1:**
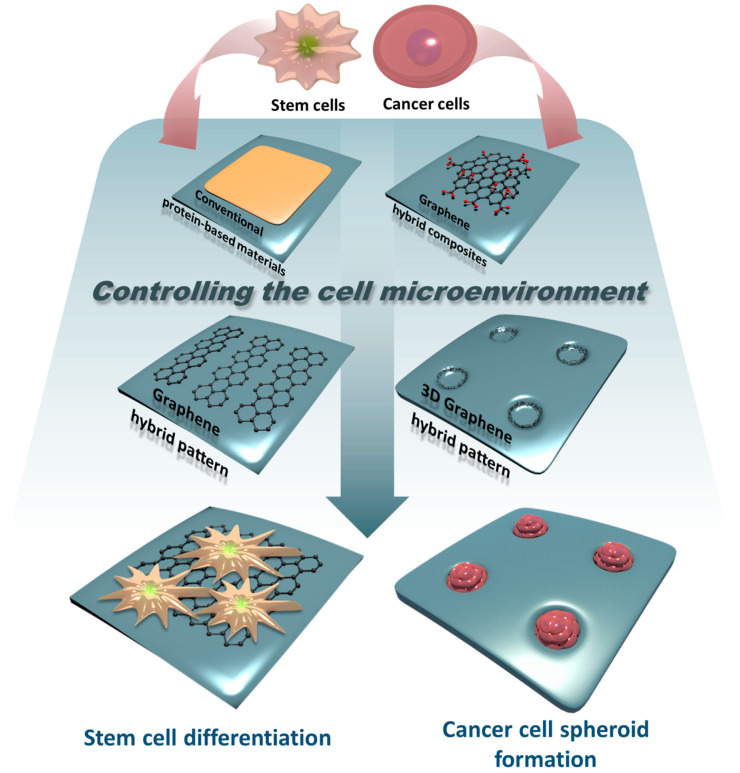
Schematic diagram related to controlling stem cell or cancer cell microenvironments using protein-based materials, graphene hybrid composites, graphene hybrid patterns, and 3D graphene hybrid patterns.

For these reasons, graphene and GO are considered to have sufficient properties to control cellular microenvironments. In fact, graphene and GO can be used in more diverse forms compared to fibrous proteins because they can form various artificial structures with other materials or by themselves relatively easily [38,45,46,47,48]. Graphene scaffolds, graphene-based hydrogels, and graphene patterns that combine graphene and polymers are related examples. The purpose of using these for controlling the cellular microenvironment is not significantly different from that of using ECM proteins, for example, controlling tumor cell growth, or stem cell differentiation. However, graphene derivatives are easy to fabricate, economical, and highly stable in extreme environments, all of which are hard to satisfy with protein-based materials. Therefore, it is meaningful to overview a myriad of platforms that incorporate graphene derivatives as a core material for regulating various cell functions and behaviors. Given this fact, in this review, we briefly introduce cellular microenvironmental control using graphene-based functional platforms (Figure 1 and Table S1). The platforms induce differentiation of different types of stem cells and are involved in cell proliferation, such as in the long-term culturing of cells. Furthermore, studies on the application of graphene-based materials to 3D cell culture methods are ongoing and will also be introduced in this review.

## 2. Controlling Cellular Microenvironments Using Conventional Protein-Based Materials

**Figure 2:**
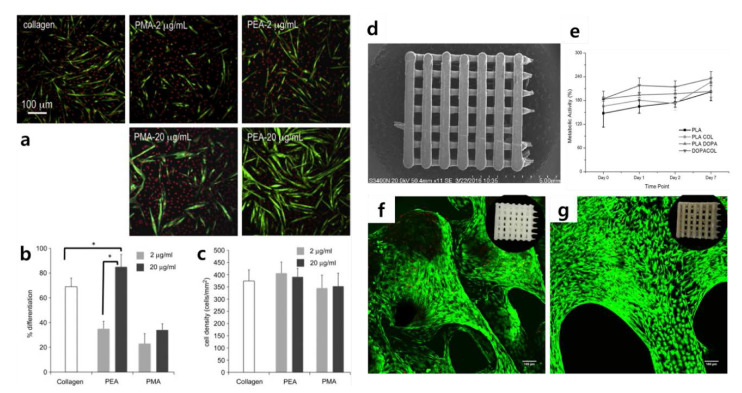
(**a**) Myogenic differentiation with different conditions. Myosin-positive cells and nuclei are marked in green and red, respectively. (**b**) Myogenic differentiation and (**c**) cell density with different FN concentrations. (**d**) Scanning electron microscopy image of poly(lactic acid) (PLA) scaffolds. (**e**) Metabolic activity of mesenchymal stem cells (MSCs) cultivated in PLA scaffolds with different coating materials. Results of Alamar Blue assay for cells cultured on (**f**) PLA scaffolds and (**g**) DOPA COL scaffolds. (**a**–**c**) The reprint of this figure from [54] is permitted by Elsevier. (**d**–**g**) The reprint of this figure from [55] is permitted by John Wiley and Sons.

To fully understand the use of graphene-based materials for controlling cellular behaviors, the control of cellular microenvironments using conventional protein-based materials should be overviewed. Standard protein-based materials are fibrous proteins, such as collagen and fibronectin, which possess excellent cell adhesion properties. They are used in many types of cell culture and assist in the proliferation of cells or induce differentiation [49,50,51,52,53]. In 2011, Salmerón-Sánchez et al. studied the effect of fibronectin and collagen on the myogenic differentiation of C2C12 myoblasts (Figure 2a–c) [50,54]. In this study, the authors dispensed fibronectin and collagen onto polymer sheets so that the myoblasts coated the polymer. The optimal coating conditions for the polymer were set by adjusting the concentration of fibronectin and collagen, and then the progress was confirmed by culturing C2C12 on the polymer sheets. They identified the myogenic differentiation of C2C12. In 2018, conventional protein-based materials were applied to control cellular microenvironments in various ways. Teixeira et al. used poly(lactic acid) (PLA) composed of collagen to bone marrow-derived stem cells (BM-MSCs) differentiation (Figure 2d–g) [55]. They devised a differentiation method for BM-MSCs using PLA scaffolds coated with polydopamine (PDA) and type I collagen. PDA functionalization has been developed as a simple, one-step approach to improve the bioactivity of biomaterial surfaces [56,57]. They were able to induce the attachment of MSCs to PLA scaffold ultimately by using collagen, which is useful for inducing cell adhesion with PDA. Teixeira et al. incubated BM-MSCs to induce osteogenic differentiation and found an effective collagen coating method for inorganic polymers.

In the study, Teixeira et al. induced the osteogenic differentiation of MSCs by treating them with collagen on the PLA scaffold structure; several similar studies have been reported [58,59,60,61,62]. However, the study by Teixeira et al. is valuable in that the researchers increased the coating efficiency for inorganic scaffolds using PDA. In addition, this study is an appropriate example of cell differentiation using conventional protein-based materials. In the case of conventional protein-based materials such as fibronectin, where stem cells are cultured after a 2D coating treatment on a general tissue culture plate, differentiation is unlikely to occur unless additional treatments are performed. In other words, cell differentiation requires physical effects that can be induced by fibrous proteins such as fibronectin. The framework that can provide this physical effect is a scaffold, as described in this study, and fibrous proteins can be absorbed into this framework and induce cell adhesion [63,64,65,66]. Taken together, it is clear that a variety of biocompatible materials (e.g., PDA, PLA) functionalized with ECM proteins (e.g., fibronectin, collagen) are effective in regulating various cellular functions, including stem cell differentiation, via controlling the cellular microenvironments.

## 3. Controlling Cellular Microenvironments Using Graphene Hybrid Composites

**Figure 3:**
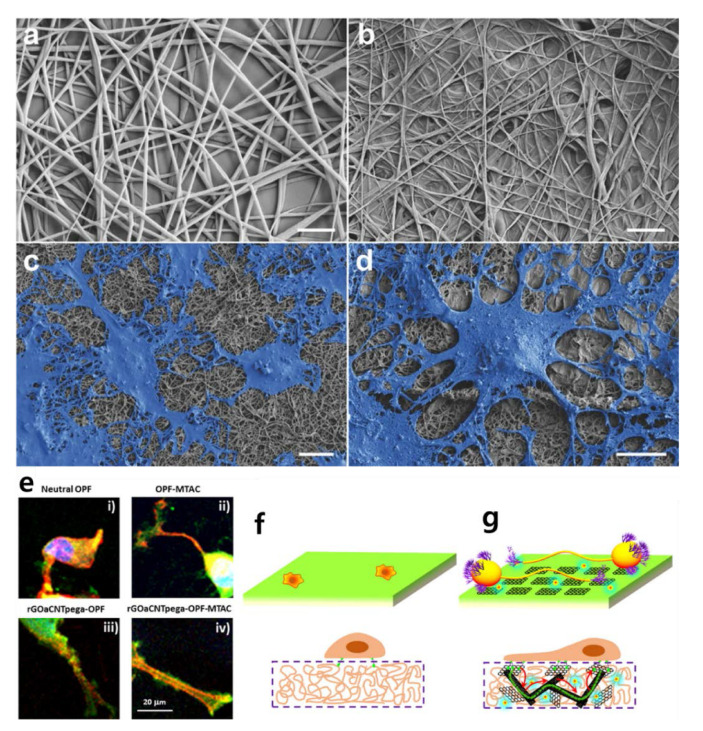
Scanning electron microscopy images of (**a**) polycaprolactone (PCL) nanofibers, (**b**) PCL nanofibers coated with graphene oxide (GO), (**c**) differentiated neural stem cells (NSCs) on PCL nanofibers, and (**d**) differentiated cells on graphene–nanofiber hybrid scaffolds. Scale bars: 2 μm. (**e**) Focal adhesion development in PC12 cells on four types of substrate. Schematic illustration of cellular behavior on (**f**) neutral OPF hydrogel and (**g**) reduced graphene oxide acrylate (rGOa)CNTpega-oligo (poly(ethylene glycol) fumarate) (OPF)-2-(methacryloyloxy)ethyltrimethylammonium chloride (MTAC) hydrogel. (**a**–**d**) The reprint of this figure from [69] is permitted by John Wiley and Sons. (**e**–**g**) The reprint of this figure from [77] is permitted by the American Chemical Society.

Research on stem cell differentiation using graphene hybrid composites has been very prolific since 2010. Considering that graphene is less cytotoxic and allows stem cells to attach and proliferate [67,68], graphene hybrid composites are actively used for stem cell differentiation, and the methods used for their application are diverse. In the early 2010s, Shah et al. succeeded in differentiating oligodendrocytes using graphene–nanofiber structures. Shah et al. developed a substrate for differentiating human neural stem cells into mature oligodendrocytes by coating graphene oxide (GO) on a nanofibrous scaffold made of polycaprolactone (PCL) (Figure 3a–d) [69]. They argued that their graphene–nanofiber hybrid scaffolds induced differentiation by providing instructive physical cues for the differentiation of oligodendrocytes without introducing differentiation inducers in the culture media. Through an approach similar to the fibrous proteins–nanofiber hybrid scaffold mentioned earlier, the differentiation of human neural stem cells (hNSCs) in this study was promoted [70,71,72].

Liu et al. developed conductive hydrogel containing carbon nanotubes and graphene oxide for nerve cell differentiation (Figure 3e–g) [73,74,75,76,77]. Specifically, a conductive hydrogel was fabricated by combining oligo (poly(ethylene glycol) fumarate) (OPF) polymer with carbon nanotubes and functionalized graphene oxide acrylate (GOa). OPF hydrogel has a positive charge, so it can play a role in nerve conduction, and the positive charges were incorporated by 2-(methacryloyloxy)ethyltrimethylammonium chloride (MTAC) to obtain rGOaCNTpega-OPF-MTAC composite hydrogel with both surface charge and electrical conductivity. Liu et al. cultivated PC12 cells on this conductive hydrogel, and not only confirmed neuronal differentiation, but also constructed an artificial nerve conduit by rolling the hydrogel into a cylindrical shape [78]. A notable feature of this study is that the conductivity of GO also applies to the hydrogel. Unlike the oligodendrocyte differentiation just mentioned, this study used the electrical properties of graphene hybrid composites. Several studies have already utilized electrical stimulation for stem cell differentiation [79,80,81], such as differentiation of PC12 cells using electrical properties of graphene hybrid composites [82,83]. 

**Figure 4:**
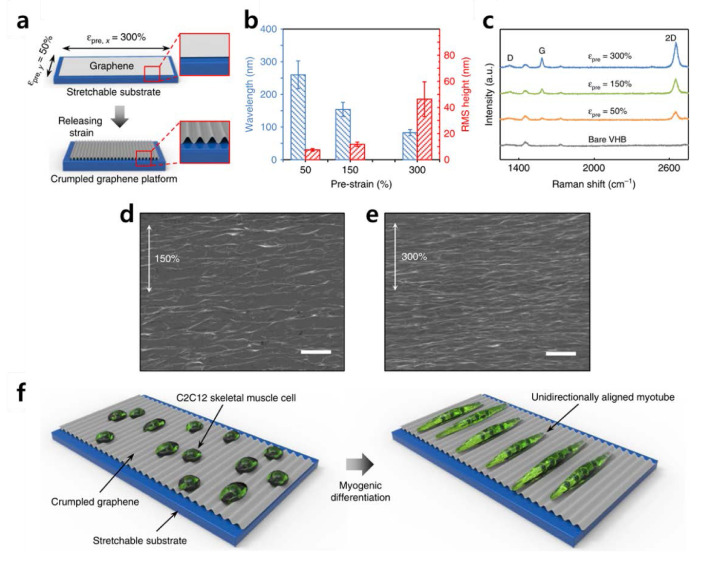
(**a**) Schematic illustration of fabricating the crumpled graphene. (**b**) Wavelength and root mean square height of crumpled graphene substrates. (**c**) Raman spectroscopy intensity of the crumpled graphene on VHB substrates by condition. (**d**–**e**) Scanning electron microscopy images of crumpled graphene fabricated from 150% and 300% prestrains. Scale bars: 1  μm. (**f**) Schematic illustrations of the myogenic differentiation of C2C12 cells on crumpled graphene. The reprint of this figure from [84] is permitted by Springer Nature.

Recently, Kim et al. induced differentiation of C2C12 mouse myoblast cells using graphene. Kim et al. produced a “crumpled grapheme” structure for cell differentiation (Figure 4) [84]. Crumpled graphene is fabricated while the tension of the stretchable substrate evenly coated with graphene is removed. C2C12 cells are cultured on the crumpled graphene to induce a myotube form. To be precise, C2C12 cells are provided with instructive physical cues by the bending of crumpled graphene. In addition, Kim et al. emphasized that the electrical properties of graphene have a profound effect on differentiation. Other studies have shown that neural stem cells or skeletal muscle cells mentioned above may promote differentiation due to electrical stimulation [85,86,87,88,89,90]. This promotion of differentiation underscores that the physical and electrical properties of graphene are useful for inducing stem cell differentiation. Taken together, it is obvious that both graphene and GO are highly advantageous for guiding stem cell differentiation, owing to the following key features: (i) the excellent absorption of ECM proteins, (ii) easy to modify on various hydrogels/polymers, (iii) highly adhesive to various cell lines, and (iv) ability to apply electrical stimulation.

## 4. Controlling Cellular Microenvironments Using Graphene Hybrid Patterns

Graphene patterns create a more deliberate physical impact beyond the control of microenvironments that can be expected from random arrangements or the electrical properties of graphene itself. An advantage is that graphene can be patterned into a specific shape or a specific arrangement to construct the cell culture type desired by researchers.

**Figure 5:**
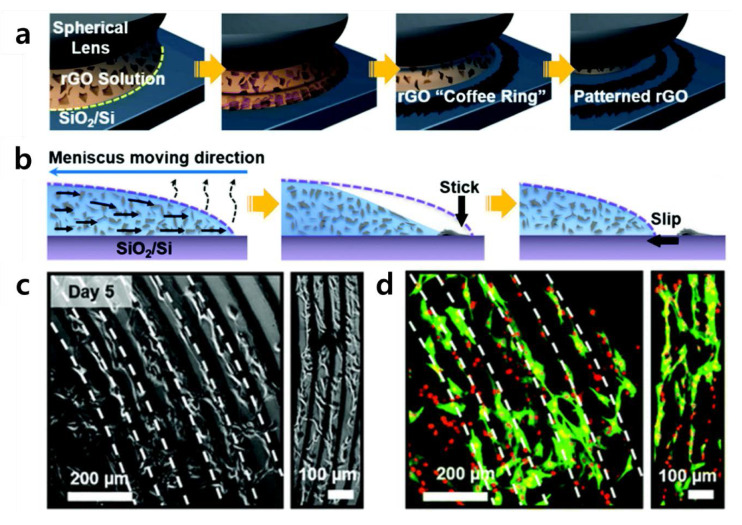
(**a**) Schematic illustration of rGO coffee-ring array manufacturing process. (**b**) Cross-sectional view of the formation of an rGO coffee-ring array. (**c**,**d**) Cell guidance effect of rGO coffee-ring array on C2C12 cells. (**c**) Optical microscopic and (**d**) fluorescence images of C2C12 cells cultured for 5 days. Alpha-smooth muscle actin (SMA) was stained with a FITC-conjugated anti-SMA antibody (green), and the cell nucleus was counterstained with PI (red). The reprint of this figure from [91] is permitted by the Royal Society of Chemistry.

Kang et al. reported a new form of GO patterning technology (Figure 5) [91,92]. They also cultivated C2C12 cells (skeletal muscle cells) on the patterns they constructed, suggesting the possibility of developing skeletal muscle on a chip. Kang et al.’s patterning method, reported as “coffee-rings”, uses a method of gradual evaporation of reduced graphene oxide (rGO) solution in contact with a spherical lens. The rGO remains on the edge of the evaporation of the solution to form a kind of ring-shaped rGO pattern, and the evaporation is repeated to pattern small diameter rings gradually. Kang et al. cultivated C2C12 cells on this pattern as noticeable contact guidance. These finely tuned rGO surfaces can explicitly elicit specific cellular responses by providing topographical and biochemical cues. From the material’s point of view, rGO has the same mechanism of cell adhesion induction as GO. However, rGO possesses functional groups such as hydroxyl and carboxyl groups, but its ability to induce cell adhesion is inferior because the number of functional groups per the same area is less than that of GO. The study by Kang et al. has some implications in that the patterned rGO arrays could be used as engineered cell-responsive environmental templates to assemble a useful tissue-on-a-chip composed of multiple types of cells, such as skeletal muscle. This study can also be compared with the study in which crumpled graphene was used, as summarized in the previous section. In both studies, the same C2C12 cell line was used, and the skeletal muscle cells were differentiated using elongated linear array culture [93,94,95,96]. Both studies were aimed at skeletal muscle cells differentiation, but there were differences in the composition of graphene.

**Figure 6:**
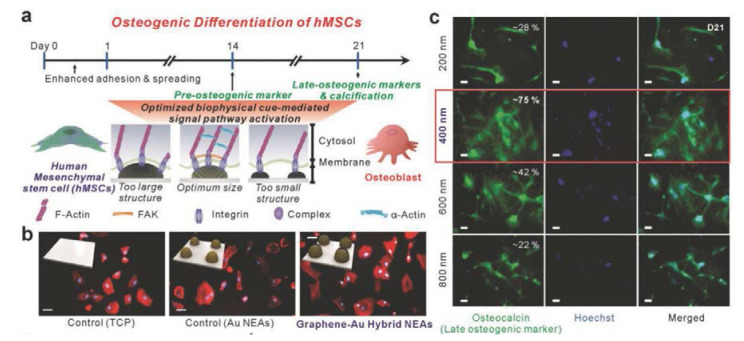
(**a**) Schematic illustration of osteogenic differentiation of human mesenchymal stem cells (hMSCs) on the graphene-Au hybrid NEAs. (**b**) Fluorescence images of hMSCs on TCP, Au NEAs, and graphene–Au hybrid NEAs substrate. (**c**) Fluorescence images of hMSCs differentiated into osteoblasts. Scale bar: 50 µm. The reprint of this figure from [98] is permitted by John Wiley and Sons.

If a graphene hybrid pattern is applied to stem cell culture, it can serve as a platform to induce their differentiation. Lee et al. produced nanoscale graphene–Au hybrid nanoarrays to induce differentiation of human mesenchymal stem cells (hMSCs) (Figure 6) [97,98]. Unlike the patterning method previously discussed in this review, Lee et al. produced a photoresistor array based on laser interference lithography (LIL). They then produced a graphene pattern on the substrate on which Au was deposited via vapor deposition (PVD) methods. Next, they cultivated hMSCs on the array and confirmed their osteogenic differentiation. In addition, the authors emphasized that a graphene–Au hybrid nanoarray platform could enhance osteogenic differentiation of hMSCs through the unique physiochemical cues from the graphene–Au hybrid nanoarray [99,100].

This study is one NSC differentiation platform using LIL, and there are many cases where LIL is applied to NSC differentiation [101,102,103,104,105,106,107]. However, Lee et al. used graphene for nanoarrays fabricated with LIL, which increased the efficiency of differentiation on their platform. In addition, a new type of graphene nanoarray platform developed using LIL has been reported. The hypothetical mechanism of the reported osteogenic differentiation of hMSCs is evidenced by the data presented. As shown in Figure 6, the difference in the degree of differentiation according to the presence or absence of the nanoarray and the presence or absence of graphene is remarkable. This finding confirms that physiochemical effects caused by nanoarrays and graphene are essential factors for osteogenic differentiation.

## 5. Controlling 3D Cancer Cell Microenvironments Using Graphene Hybrid Composites

Controlling the cellular environment using graphene is not limited to stem cells. This control can also be applied to tumor cells, as demonstrated in recent studies [108]. As previously mentioned, graphene can induce cell adhesion, so if combined with some biocompatible hydrophobic polymers, a cellular pattern is formed. In particular, studies are being conducted to produce 3D cultures of tumor cells by applying this pattern to tumor cells. Unlike studies on stem cells, numerous studies on tumor cells are specialized in topics such as drug delivery to inhibit/induce growth of tumor cells or regulate growth. Therefore, research has been focused on the development of a 3D cell culture method similar to tumor formation in the human body, and research for improving the efficiency of the culture method is currently underway.

**Figure 7:**
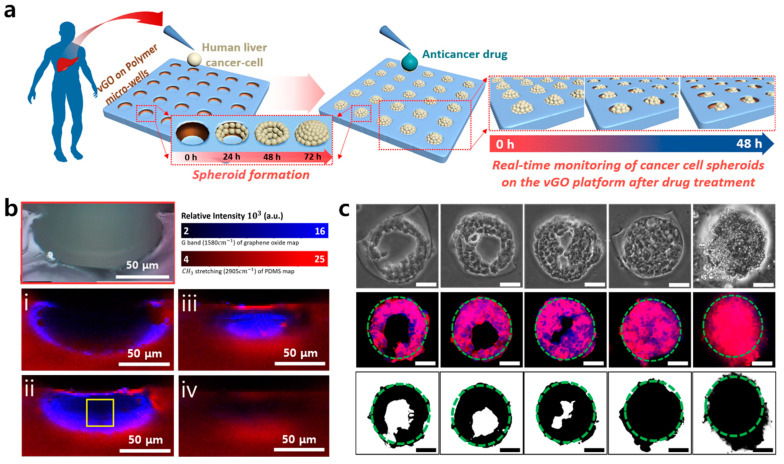
The characterization of the vertically coated graphene oxide micropattern (vGO-MP) platform. (**a**) Schematic illustration showing cancer cell spheroid formation on vGO-MP and anticancer drug screening process of cancer spheroids on the vGO-MP. (**b**) Raman mapping images of micro-well cross-section by focal point. (**c**) Optical microscopic images (top row), fluorescence images (middle row), and computer-based binary images (bottom row) of a HepG2 spheroid in a vGO-MP. Scale bars: 50 μm. The reprint of this figure from [109] is permitted by John Wiley and Sons.

Cancer cell microenvironmental control using graphene has also been applied to a 3D cell culture method. Kim et al. produced a graphene oxide-based platform and reported HepG2 cell spheroid formation through cell proliferation (Figure 7) [109]. The platform was built using a graphene vertical coating technology they developed, and the overall effect of this technology was to induce spheroid formation in tumor cells by coating graphene oxide on the walls of a polydimethylsiloxane (PDMS) micro-well. Using this coating technology, which can pattern graphene oxide on a specific surface using moisture treatment with GO [110,111,112], it is possible to induce cells to preferentially adhere to the wall of the PDMS micro-well. They photographed micro-well monolayers via Raman spectroscopy to identify vertical graphene patterns coated on the wall [113,114]. Cells attached to the wall of the spherical micro-well begin to fill the wells time-dependently and eventually form a tumor cell spheroid inside the well. In particular, Kim et al. also implemented donut-like spheroid formation in which cells attached to the wall surfaces of a micro-well coated with graphene oxide. This study suggested the induction of tumor cell spheroid formation through cell proliferation and a new form of spheroid formation.

**Figure 8:**
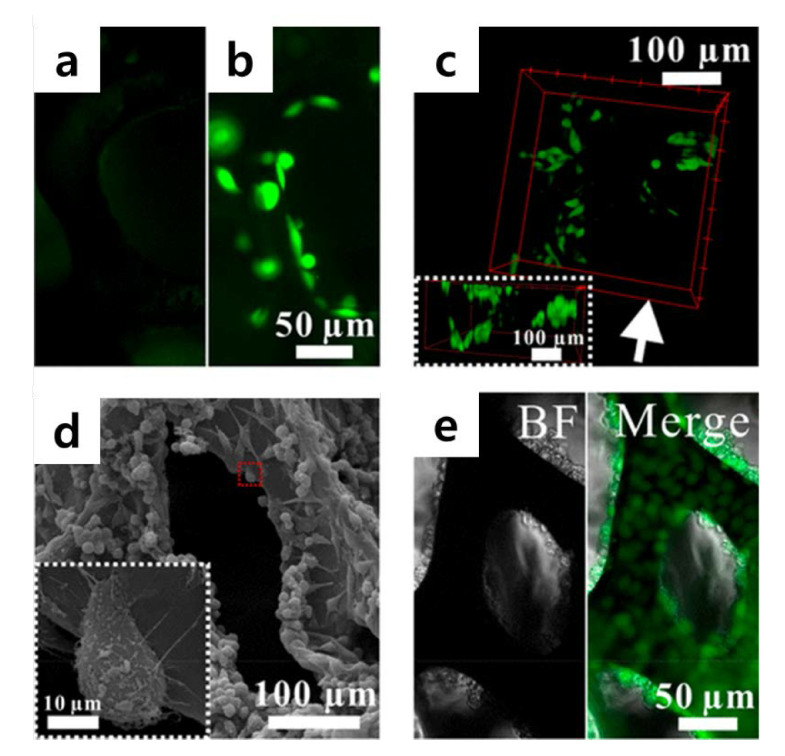
(**a**) Fluorescence images of GFP-HeLa cells cultured on (**a**) GF, (**b**) GF/ phenylboronic acid (PBA)/3-aminophenylboronic acid (APBA), and (**c**) GF/PBA/APBA composites. (**d**) SEM images of HeLa cells cultured on GF/PBA/APBA composites. The enlarged image of the red rectangle area is shown in a white square. (**e**) Microscopic images of HeLa cells cultured for 11 days. The bright field (BF) and the merger of the bright field and fluorescence field (Merge) images are presented. The reprint of this figure from [115] is permitted by the American Chemical Society. (APBA), and (PBA).

Hu et al. devised a slightly different approach to control 3D cellular microenvironments (Figure 8) [115]. They used a graphene-based 3D scaffold for cell culture and conducted a study to control the cellular microenvironment. Recently, several studies have been undertaken to reproduce the in vivo environment in vitro using a 3D scaffold. Hu et al. introduced a graphene-based 3D scaffold platform that can be used for long-term cell culture and that enables real-time electrochemical monitoring. Specifically, the platform is composed of functionalized graphene foam, 3-aminophenylboronic acid (APBA), and phenylboronic acid (PBA), wherein APBA was used as a small-adhesive molecule to control cell adhesion [116,117,118]. This control allowed their 3D scaffold to become a cytocompatible surface for cell adhesion and proliferation, resulting in long-term cell culture.

Recently, Wan et al. also reported that GO exerts a positive effect on the growth of cancer cells when incorporated with the scaffold structure [119]. In this study, a scaffold consisting of cellulose acetate (CA) microfibers in combination with bacterial cellulose (BC) was fabricated, and GO was further added to the CA-BC scaffold. Using a GO-modified CA-BC scaffold, several cellular functions including cell adhesion, migration, and growth were assessed. Interestingly, Wan et al. confirmed that higher cell growth was observed in the GO/CA-BC scaffold than in typical CA-BC hydrogel based on results of colorimetric assay (CCK-8 method) and optical image monitoring. Finally, they reported that the activity of human breast cancer cells cultured in a GO/CA-BC scaffold was significantly higher, mostly owing to the increase in spreading of the cells attached on GO.

## 6. Conclusions

Through the studies discussed above, we divided cellular microenvironmental control using graphene into two main categories. One is ‘cellular microenvironments of stem cells’, and the other is ‘cancer cell microenvironments’. These cellular microenvironments were controlled using various types of graphene-based platforms. In the case of stem cells, differentiation was induced or promoted. In the case of cancer cells, proliferation was adjusted to enable the formation of 3D spheroids of cancer cells and long-term cell culture. Graphene derivatives regulate the microenvironments of target cells in various ways. Specifically, the induction of differentiation of hNSCs has been demonstrated using graphene–polymer scaffolds, and the control of HeLa cell attachment and growth using graphene platforms such as crumpled graphene, and graphene patterning are notable. In addition, the GO vertical patterning method in which graphene is three-dimensionally patterned on the side wall of micro-well arrays, has provided a new platform for an effective method for 3D culture of cancer cells. All of these “controlling cellular microenvironments” approaches are enabled due to distinct physical and electrical properties of graphene including excellent cell adhesion, flexibility in physical transformations, and stability against physical/chemical stimuli. Such characteristics of graphene and GO are known to affect various biological phenomena including (i) the absorption or repulsion of growth/differentiation factors on the surface, (ii) ECM protein alignment, and (iii) anchoring of integrin receptors on the cell membrane to the artificial surface. These physicochemical properties of GO allow a limitation or enhancement of cell adhesion and spreading, which ultimately lead to the changes in various cell functions (e.g., growth, migration, differentiation) via alterations in the upstream cytoskeletal dynamics and downstream gene expression. Broadening our horizons, we were able to determine the positive changes that graphene-based materials and platforms will have on regenerative and clinical medicine in the future. In particular, graphene-modified hydrogels and graphene patterns can be used to achieve cells of interest from various stem cell sources with high conversion efficiency, which will ultimately accelerate the use of stem cells for regenerative therapies. In the case of cancer research, graphene hybrid materials, including graphene-coated micro-wells, can be used to restrict cell adhesion and growth through generation of 3D tumor spheroid arrays and further rapid anticancer drug screening. Taken together, we can conclude that controlling cellular microenvironments using graphene-based materials is highly promising for both stem cell-based regenerative medicine and cancer research.

## Supplementary Materials

The following are available online at https://www.mdpi.com/1996-1944/13/18/4008/s1, Table S1: Conventional protein-based materials and graphene-based material platforms for controlling cellular microenvironments.
