# Supplementary material for: Graphene Hybrid Materials for Controlling Cellular Microenvironments
Source: Materials (Basel). 2020 Sep 10;13(18):4008. doi: 10.3390/ma13184008 (PMC7559936; doi:10.3390/ma13184008)
Supplement: Supplementary file 1 [file materials-13-04008-s001.pdf]

## Supplementary Materials:

**Table S1.** Conventional protein-based materials and graphene-based material platforms for controlling cellular microenvironments.

| Materials | Structure | Target Cells | Purpose | Ref. |
|---|---|---|---|---|
| Fibronectin, collagen | - | Mouse myoblast cells | Myogenic differentiation | [1] |
| Collagen, PLA, PDA | Scaffold | Human mesenchymal stem cells | Osteogenic differentiation | [2] |
| Graphene oxide | Nanofibrous scaffold | Human mesenchymal stem cells | Oligodendrocyte differentiation | [3] |
| Graphene oxide | Nanofibrous scaffold | Rat pheochromocytoma cells | Neuronal differentiation | [4] |
| Graphene oxide | Crumpled graphene | Mouse myoblast cells | Myogenic differentiation | [5] |
| Reduced graphene oxide | Coffee-ring array | Mouse myoblast cells | Myogenic differentiation | [6] |
| Graphene | Graphene–Au hybrid nanoarray | Human mesenchymal stem cells | Osteoblast differentiation | [7] |
| Graphene oxide | Vertically coated GO micropattern | Human liver cancer cells | 3D cell culture | [8] |
| Graphene | Nanofibrous scaffold | Human cervical cancer cell | Long-term cell culture | [9] |
| Graphene oxide | Nanofibrous scaffold | Human breast cancer cell | Induce cell proliferation | [10] |

## References


1. Salmerón-Sánchez, M.; Rico, P.; Moratal, D.; Lee, T.T.; Schwarzbauer, J.E.; García, A.J. Role of material-driven fibronectin fibrillogenesis in cell differentiation. *Biomaterials* **2011**, *32*, 2099–2105, doi:10.1016/j.biomaterials.2010.11.057.
2. Teixeira, B.N.; Aprile, P.; Mendonça, R.H.; Kelly, D.J.; Thiré, R.M.D.S.M. Evaluation of bone marrow stem cell response to PLA scaffolds manufactured by 3D printing and coated with polydopa-mine and type I collagen. *J. Biomed. Mater. Res. Part B Appl. Biomater.* **2019**,*107*, 37–49.
3. Shah, S.; Yin, P.T.; Uehara, T.M.; Chueng, S.T.D.; Yang, L.; Lee, K.B. Guiding stem cell differ-entiation into oligodendrocytes using graphene-nanofiber hybrid scaffolds. *Adv. Mater.* **2014**, *26*, 3673–3680.
4. Liu, X.; Miller, A.L.; Park, S.; Waletzki, B.E.; Zhou, Z.; Terzic, A.; Lu, L. Functionalized Carbon Nanotube and Graphene Oxide Embedded Electrically Conductive Hydrogel Synergistically Stimulates Nerve Cell Differentiation. *ACS Appl. Mater. Interfaces* **2017**, *9*, 14677–14690, doi:10.1021/acsami.7b02072.
5. Kim, J.; Leem, J.; Kim, H.N.; Kang, P.; Choi, J.; Haque, F.; Kang, D.; Nam, S. Uniaxially crumpled graphene as a platform for guided myotube formation. *Microsyst. Nanoeng.* **2019**, *5*, 53–10, doi:10.1038/s41378-019-0098-6.
6. Kang, S.H.; Shin, Y.C.; Hwang, E.Y.; Lee, J.H.; Kim, C.-S.; Lin, Z.; Hur, S.H.; Han, D.-W.; Hong, S.W. Engineered "coffee-rings" of reduced graphene oxide as ultrathin contact guidance to enable patterning of living cells. *Mater. Horiz.* **2019**, *6*, 1066–1079, doi:10.1039/c8mh01381k.
7. Lee, J.-H.; Choi, H.K.; Yang, L.; Chueng, S.-T.D.; Choi, J.-W.; Lee, K.-B. Nondestructive Real-Time Monitoring of Enhanced Stem Cell Differentiation Using a Graphene-Au Hybrid Nanoelectrode Array. *Adv. Mater.* **2018**, *30*, e1802762, doi:10.1002/adma.201802762.
8. Kim, C.-H.; Suhito, I.R.; Angeline, N.; Han, Y.; Son, H.; Luo, Z.; Kim, T.-H. Vertically Coated Graphene Oxide Micro-Well Arrays for Highly Efficient Cancer Spheroid Formation and Drug Screening. *Adv. Healthc. Mater.* **2020**, *9*, e1901751, doi:10.1002/adhm.201901751.
9. Hu, X.-B.; Liu, Y.-L.; Wang, W.-J.; Zhang, H.-W.; Qin, Y.; Guo, S.; Zhang, X.-W.; Fu, L.; Huang, W.-H. Biomimetic graphene-based 3D scaffold for long-term cell culture and real-time electro-chemical monitoring. *Anal. Chem.* **2018**, *90*, 1136–1141.
10. Wan, Y.; Lin, Z.; Zhang, Q.; Gan, D.; Gama, M.; Tu, J.; Luo, H. Incorporating graphene oxide into biomimetic nano-microfibrous cellulose scaffolds for enhanced breast cancer cell behavior. *Cellulose* **2020**, *27*, 4471–4485, doi:10.1007/s10570-020-03078-w.
